# The effectiveness of exercise as a treatment for postnatal depression: study protocol

**DOI:** 10.1186/1471-2393-12-45

**Published:** 2012-06-09

**Authors:** Amanda J Daley, Kate Jolly, Debbie J Sharp, Katrina M Turner, Ruth V Blamey, Sarah Coleman, Mary McGuinness, Andrea K Roalfe, Ian Jones, Christine MacArthur

**Affiliations:** 1 Primary Care Clinical Sciences, School of Health and Population Sciences, University of Birmingham, Birmingham, UK; 2 Unit of Public Health, Epidemiology and Biostatics, School of Health and Population Sciences, University of Birmingham, Birmingham, UK; 3 Academic Unit of Primary Health Care, University of Bristol, Bristol, UK; 4 Perinatal Mental Health Service, The Barberry, Birmingham and Solihull Mental Health Foundation Trust, Birmingham, UK; 5 Institute of Psychological Medicine & Clinical Neurology, Cardiff University, Cardiff, Wales, UK; 6 Primary Care Clinical Sciences, University of Birmingham, Birmingham, B15 2TT, UK

**Keywords:** Exercise, Postnatal depression

## Abstract

**Background:**

Postnatal depression can have a substantial impact on the woman, the child and family as a whole. Thus, there is a need to examine different ways of helping women experiencing postnatal depression; encouraging them to exercise may be one way. A meta analysis found some support for exercise as an adjunctive treatment for postnatal depression but the methodological inadequacy of the few small studies included means that it is uncertain whether exercise reduces symptoms of postnatal depression. We aim to determine whether a pragmatic exercise intervention that involves one-to-one personalised exercise consultations and telephone support plus usual care in women with postnatal depression, is superior to usual care only, in reducing symptoms of postnatal depression.

**Methods:**

We aim to recruit 208 women with postnatal depression in the West Midlands. Recently delivered women who meet the ICD-10 diagnosis for depression will be randomised to usual care plus exercise or usual care only. The exercise intervention will be delivered over 6 months. The primary outcome measure is difference in mean Edinburgh Postnatal Depression Scale score between the groups at six month follow-up. Outcome measures will be assessed at baseline and at six and 12 month post randomisation.

**Discussion:**

Findings from the research will inform future clinical guidance on antenatal and postnatal mental health, as well as inform practitioners working with postnatal depression.

**Trial registration number:**

ISRCTN84245563

## Background

It is estimated that depression will be the second most common cause of disability worldwide by 2020 [1]. Postnatal depression (PND) is a serious problem across cultures and affects about 10-15% of women some time in the year after giving birth [2,3]. Women with postnatal depression can experience disabling symptoms of low mood, irritability, fatigue, insomnia, changes in appetite, anxiety, guilt, inability to cope, feelings of worthlessness and thoughts of suicide. Frequently exacerbating these symptoms are low self-esteem, lack of confidence, and unrealistic expectations of motherhood. Women who have PND are twice as likely to experience subsequent episodes of depression in later life [4]. PND has health consequences not only for the mother but also for the child and family as a whole. Cognitive and emotional development and social behaviour have been shown to be adversely affected in children whose mothers have PND [5]. PND can cause impaired maternal-infant interactions and negative perceptions of infant behaviour [6]. Marital difficulties are not uncommon and the partner may also become depressed [7]. Suicide is a rare but devastating consequence of PND.

After giving birth, many women have excess weight and decreased fitness levels [8,9]. New mothers have reported weight gain to be a significant concern for them [10]. Studies of pregnant and postpartum women have indicated they are at high risk for inactivity and reductions in previously established levels of activity [11]. These health concerns would also apply to women with PND. The new physical activity guidelines “Start Active Stay Active” published by the Chief Medical Officers in the UK [12] state that participation in physical activity can have an important role in promoting mental health and well-being. Meta-analyses and reviews [13-15] have concluded that exercise may be effective in reducing depression in adult populations, although concerns have been raised about the methodological quality of trials to date. On the basis of the available evidence in 2009 [16] the National Institute for Health and Clinical Excellence (NICE) in England recommended that people with persistent sub-threshold depressive symptoms or mild to moderate depression should be advised of the benefits of exercise.

Based on evidence of the positive effects of exercise on depression in general populations it seems plausible that regular exercise may also be an effective intervention for the treatment of PND. However, direct extrapolation from research with general populations to those experiencing PND is not necessarily appropriate because the circumstances of early motherhood are sufficiently different to preclude this. Based on general population evidence and findings from two very small trials (total n = 39) that recruited women with PND, in 2006 NICE [17] recommended in their guidance on the management of antenatal and postnatal mental health, that health professionals should consider exercise as a treatment for PND.

A systematic review and meta-analysis [18] of randomised and quasi randomised controlled trials has examined the effects of exercise on PND. Five trials (six reports) [19-24] were identified (total N: n = 114 intervention; n = 107 comparators). In three trials, participants were receiving antidepressant medication and/or psychological therapies, one trial excluded participants if they were currently using antidepressants or receiving psychotherapy and one did not provide this information. When compared with no-exercise, exercise significantly reduced symptoms of PND (weighted mean difference in Edinburgh Postnatal Depression Scale [25] score was −4.00 points (95% CI: -7.64 to −0.35)) (standardised mean difference: -0.81 (95% CI: -1.53 to −0.10)). Whilst the magnitude of this effect might appear initially encouraging, caution is required when interpreting this finding because significant heterogeneity was found and because the effect size was reduced considerably (and became non-significant) when the trial [20] that included exercise as a co-intervention with social support was excluded (standardised mean difference = −0.42 (CI:-0.90 to 0.05)). Heterogeneity was no longer present either. Therefore it is uncertain whether exercise reduces symptoms of PND and the significant effect size of −0.81 is contingent on the inclusion of one trial where exercise was a co-intervention. We should also remain cognisant that trials were small, confidence intervals were wide, had limited follow-up and included samples of volunteers and not clinically defined cases according to diagnostic criteria. There were no studies on long term effectiveness. Given the paucity of high quality research, it is necessary to evaluate more fully the effectiveness of exercise as a treatment for PND.

In summary, a large trial that compares exercise with standard treatment(s) and which includes longer term follow-up is now essential given the recommendation from NICE [17] that health professionals should consider exercise as treatment for PND. We are aware of no ongoing trials in this field and therefore the proposed research has the potential to make a significant contribution to the literature. No previous research has investigated the views of depressed postnatal women participating in exercise programmes and a qualitative study will be nested within this trial. The qualitative study will aid our understanding why exercise, in addition to usual care, was or was not superior to usual care in reducing symptoms of PND. These insights could subsequently inform clinical practice and future interventions with women experiencing PND.

## Aims and Objectives

The primary aim of the study is to determine whether usual care plus a pragmatic exercise intervention is superior to usual care only, in reducing PND. The secondary aim is to compare physical activity, quality of life, body weight, BMI, body image and vitality scores between those receiving the exercise intervention plus usual care with those receiving usual care only. A qualitative study will be nested within the trial to explore the acceptability of the intervention to newly delivered women and to illuminate possible reasons for the quantitative findings.

## Methods/Design

### Design and population

This study is a pragmatic two arm randomised controlled trial (individual randomisation) with participants allocated to usual care plus exercise or usual care only. We aim to recruit 208 women with clinically defined postnatal depression (ICD-10 diagnosis of depression). Recruitment will take place between April 2010 and January 2012.

### Inclusion and exclusion criteria

#### Inclusion criteria

· An ICD-10 diagnosis of depression, following screening using the EPDS at 10–14 and 12–16 weeks and CIS-R at 13–17 weeks after giving birth. Women with a mixed diagnosis of anxiety and depression are also eligible

· Aged 18 years or more

· Currently inactive (defined as not meeting the current public health guidelines for physical activity (i.e. <150 minutes of moderate intensity physical activity per week in previous 7 days))

#### Exclusion criteria

· Pregnant at baseline

· Experiencing psychotic symptoms at baseline

· Dependent on illicit drugs or alcohol

· Not proficient in English at a level to complete research assessments

· GP considers patient unsuitable for the trial

· Women whose babies have died

· Women not living with the baby

#### Recruitment of women

##### Patient identification and screening (10–14 weeks after birth)

Recently delivered women who live within South Birmingham and Birmingham East and North Primary Care Trusts (PCTs) are identified from the computerised Child Health System (CHS). This approach allows us to systematically identify all women who have recently given birth and which practices they are registered with in these PCTs. Representatives from the CHS complete searches of new births every two weeks throughout the recruitment period.

**Figure 1:**
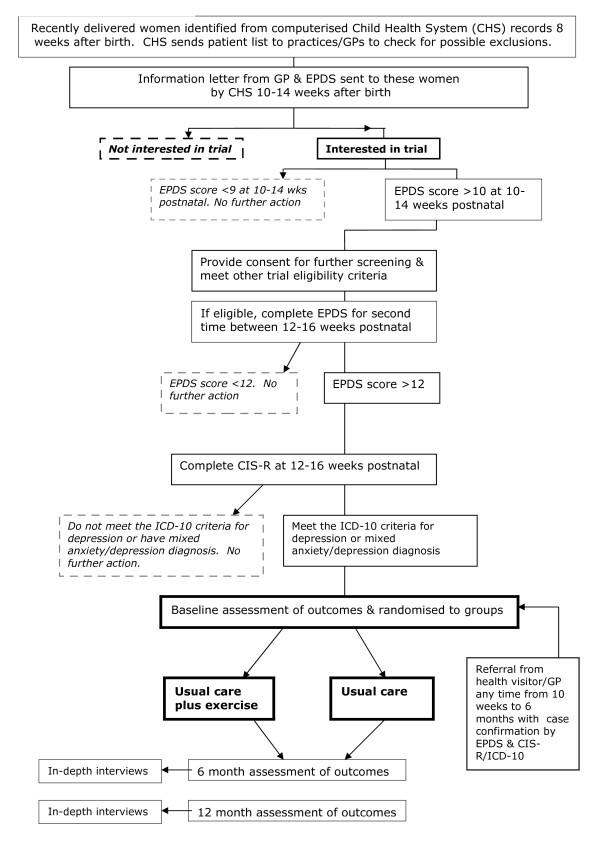
Trial Flow Diagram.

Participating GPs/practices are asked to confirm exclusions from the CHS patient lists of new births. Once exclusions are made the CHS mail the trial information letter (on GP practice headed paper) and the Edinburgh Postnatal Depression Scale [25] (EPDS) to women on behalf of the GP/practice. Women are sent this at approximately 10–14 weeks after giving birth and asked to return their completed EPDS to the research team using a prepaid envelope. Non-responders are sent a reminder two weeks later. A brief written overview of the trial is given to women at this stage, but written informed consent is obtained only for the research team to contact women who score >10 on this first EPDS to arrange completion of the EPDS for a second time and the Clinical Interview Schedule-Revised [26] 12–16 weeks after birth (see below). See Figure 1 for trial flow diagram.

##### Case definition (home visit at 12–16 weeks after birth)

In order to rule out the possibility of transient depression, women who score >10 on the first EPDS at 10–14 weeks complete a second EPDS about two weeks later at 12–16 weeks by telephone. Women who score >12 on the second EPDS at 12–16 weeks complete a Clinical Interview Schedule-Revised [26] (CIS-R) at a home visit. Based on the information obtained in the CIS-R, women are assessed as being a case according to the ICD-10 depression criteria. Women with a mixed diagnosis of anxiety and depression are also considered a case.

The trial participant information sheet is sent to women at least 2 days prior to the CIS-R home visit and women have already received an information letter from their GP. Women are given the opportunity to ask questions during the home visit before providing written informed consent for the trial. Eligible women complete the baseline questionnaires at the home visit, prior to randomisation.

##### Referral from GPs and health visitors/late onset

Women who present with depression after 10–14 weeks but within 6 months of giving birth are able to enter the trial via referral from their GP or health visitor. Women who are referred in this way have to complete the EPDS twice (as described earlier), the CIS-R, meet the ICD-10 criteria for depression, as well as other trial eligibility criteria as described. Women with a mixed diagnosis of anxiety and depression are also eligible via this route.

##### Baby clinics

In addition to the recruitment strategies outlined above, study leaflets are distributed to women when they attend their practice for baby clinic/check. Baby clinics usually take place at 8, 12 and 16 weeks after birth in England. If women indicate an interest in the study as a result of reading the leaflet they are asked to complete the EPDS screening questionnaire and reply form. These women complete all the screening processes as previously described and have to meet the ICD-10 criteria for depression.

#### Outcomes and allocation of participants to trial groups

##### Primary outcome

The primary outcome measure is the difference in mean EPDS [25] score between the groups at 6 month follow-up (end of the intervention).

#### Secondary outcomes

Quality of life is determined using the SF-12 [27] and EQ-5D questionnaire [28]. Body image is assessed using subscales from the Maternal Adjustment and Maternal Attitudes Questionnaire (MAMA) [29]. Vitality is measured using the Subjective Vitality Scale [30]. Perceived social support and social networks are assessed using items from the 2000 Office of National Statistics Survey of the mental health of households in Great Britain [31]. The Social Support for Physical Activity Scale [32] and the Self-efficacy Scale for Exercise [33] are completed by participants. Physical activity is measured objectively using accelerometers (in a 50% random sub-sample) and subjectively using the IPAQ-short [34]. Body weight and height are assessed objectively. Adverse events resulting from the intervention are systematically recorded.

#### Assessment of outcomes and blinding

A distant internet randomisation service is used to allocate women to the trial groups. The research team informs women of their allocation after completion of the baseline assessment at the home visit. The randomisation is stratified by baseline EPDS score (13–16 or ≥16). At baseline we collect demographic data; this includes age, marital status, ethnicity, social class, parity, method(s) of infant feeding, history of psychiatric illness, referral(s) to psychiatric and/or mental health services, mode of delivery, level of social support, current medication and pregnancy and antenatal exercise patterns as well as all the outcome measures.

Outcome measures will be assessed at baseline, six months (immediately post intervention) (primary) and 12 months post randomisation. At baseline, the questionnaires (see exception below) are completed and weight is assessed at the home visit. Those randomised to wear the actiheart have the device fitted at the home visit and are asked to wear it for three consecutive days. The research team collect the actiheart from participants and the IPAQ is then completed.

At follow ups, the questionnaires are mailed to all participants at the appropriate time and collected by home visits from the research team. Weight is also measured at the follow up home visits. Those wearing the actiheart have the device fitted at the home visits, are asked to wear it for 3 consecutive days and then complete the IPAQ.

#### Process outcomes/adherence

Participants randomised to the exercise intervention group are asked to complete exercise diary logs that detail the dose/amount and type of exercise completed during weeks 4, 8, 12, 16, 20 of the intervention. Logs are completed with the PAF either in person or over the phone or sent and returned by post.

### Sample size

A sample of 83 patients randomised to each group will be sufficient to detect a 1.95 unit difference (effect size = 0.5 standard deviations) in EPDS score between the groups at the 6 month follow-up with 90% power, 5% significance level. The numbers required increase to 104 in each arm when allowance is made for a 20% dropout rate at 6 month follow-up (n = 208). A sample size of 208 would also be sufficient to detect a 1.76 unit difference (effect size 0.45) in EPDS score between the groups at 6 month follow-up with 80% power, 5% significance level. We would expect maximum impact at the end of the intervention and exercise participation to tail off over time; therefore six months has been selected as the primary follow-up point.

### Analysis plan

Baseline characteristics of participants will be tabulated by randomisation arm. Primary analysis will compare mean EPDS score at the 6 months follow-up between trial groups, adjusting for baseline scores using analysis of covariance. Similar analyses will be conducted for the secondary outcomes. Secondary analyses will adjust for covariates (e.g. age, gender, initial weight, ethnicity, education level) again using analysis of covariance. To explore any longer term effects, a repeated measures mixed model analysis of the primary and secondary outcomes will be undertaken, comparing groups across the 6 and 12 month time points. EPDS score taken at the home visit will be used as the baseline score in analyses. We will also report data on the proportion of women in each group who score less than 13 points on the EPDS at follow-up. All analysis will be by intention to treat.

### Intervention

#### Rationale

In developing the exercise intervention consideration was given to the Cochrane review [35] of physical activity interventions which reported a greater consistency of effect estimates for those studies where there were four or more contacts between those delivering the intervention and participants, compared to studies where there were less than four contacts. This review also showed that a mixture of professional guidance and self-direction plus on-going support leads to more consistent effect estimates. The intervention will use the processes of change from the Transtheoretical Model conceptual framework [36] as vehicles to promote and facilitate exercise behaviour change.

Different types of exercise interventions have been used in previous trials involving depressed postnatal women as well as other depressed populations, including centre/supervised classes, community based group pram walks and home based exercise. Whilst all have been shown merit, home based exercise interventions are attractive because they are low cost. For women with PND, complications such as childcare responsibilities, fatigue and breastfeeding routines may reduce their opportunities and enthusiasm for exercise [37], particularly if they involve significant travel time or are offered at specific times of the day. Thus, any programme that promotes exercise in this population will need to take account of such factors. It is for these reasons that we have chosen to offer women a home/community based exercise intervention, which has potential to be implemented in different primary care contexts, if shown to be effective. A home-based programme involving consultations and phone calls was also found to be feasible in our pilot trial [19], where the exercise group reported more moderate intensity exercise and significantly higher self-efficacy for exercise than usual care.

Six months has been selected as the intervention duration as this is likely to be the maximum time period for which a pragmatic, low cost intervention can be viable.

#### Behavioural goal of the intervention

While current public health guidelines suggest that adults should achieve at least 30 minutes of moderate intensity exercise on at least 5 days per week [12] few people meet this target currently and this target may not be initially desirable or achievable in women who are depressed after childbirth. Therefore, the intervention goal is progressive over time. The initial goal of the interventions (weeks 1–12) is for participants to progress towards accumulating 30 minutes of moderate intensity exercise on three days per week. During weeks 13–24, the intervention encourages participants to work towards accumulating 30 minutes of moderate intensity exercise on 3–5 days per week, in broad agreement with current public health guidelines [12]. Women are encouraged to first focus on the frequency of their exercise, and then duration.

#### Overview of the exercise intervention

The exercise intervention lasts six months. As used in our feasibility pilot trial [19] the intervention involves two one-to-one personalised exercise consultations (during months 1 and 2), telephone calls (during months 3 and 4) that promote exercise. In addition, participants are mailed information leaflets throughout the 6 month intervention period. The intervention components are described in more detail below.

#### Consultations and telephone contact

The personalised consultations are centred on equipping women with the skills, knowledge and confidence needed to participate in regular exercise and delivered by a PAF in participants’ home or general practice. Consultations last about 40–60 min. The first consultation focuses on uptake of exercise and on enhancing motivation, self-efficacy for exercise, overcoming barriers and developing appropriate activity goals. Participants are given a pedometer as a motivational aid. Where possible, a short 'walk and talk' (with or without the baby) is incorporated within the first consultation so that practical issues such as perceived exertion monitoring and exercise safety can be explored with women. Participants are given information about local opportunities to exercise; including details of any postnatal specific exercise/walking groups. Four/five weeks after this, participants have a second consultation centred on the prevention of relapse back to sedentary behaviour and/or improving maintenance of an active lifestyle.

Follow-up telephone calls lasting about 15–20 minutes are made during months three and four of the intervention. The focus of the calls is the prevention of relapse back to sedentary behaviour and/or improving maintenance of an active lifestyle.

#### Leaflets

Participants receive four information leaflets in months 3, 4, 5 and 6. The purpose of the prompts is to further encourage and prompt participation in exercise.

#### Intervention quality control procedures

The PAF are trained to deliver the exercise consultations and support phone calls following established guidelines [38,39] and following a written standard manual. A random sample of consultations and telephone calls are tape recorded and assessed for fidelity by an independent researcher.

#### Usual care

There is no interference with usual care. The GP and patient can decide on any additional treatments. The usual care only group is sent the study “Looking after yourself” leaflet at baseline and exercise is not further encouraged beyond receipt of this single leaflet. Women in the usual care group will be offered an exercise consultation at the end of their involvement in the study.

### Qualitative study

In-depth interviews are held with trial participants, purposively sampled to ensure they include individuals in both arms of the trial and who vary in terms of their baseline EPDS scores. Within this sampling approach, we aim for maximum variation in relation to age, ethnicity, social class and parity. About 25 participants will be interviewed in total, i.e. 15 from the intervention group and 10 from the control group, the final number depending on when data saturation is reached. Each participant is interviewed on two occasions, i.e. once the primary and final outcome measures have been completed at 6 and 12 months post-randomisation, so that data can be used to illuminate possible reasons for quantitative findings at these two time points. The longitudinal design will allow assessment of how women’s views and experiences change over time and, for those in the intervention arm, the extent to which changes made whilst receiving the intervention are maintained once support has ended. The 6 month interview is held on a face-to-face basis and explores participants’ views and experiences of PND, the trial and usual care. The 12 month interview is shorter, held by telephone, and focuses on the participant’s experiences in the second half of the trial, in terms of their symptoms and approaches to managing their PND.

## Discussion

Approximately 800,000 women give birth each year in the UK. The prevalence of PND is reported to be about 10-15%, indicating approximately 80–120,000 women may develop PND each year. On the basis of very limited evidence NICE in 2007 [17] recommended in their guidance on the management of antenatal and postnatal mental health that exercise should be considered as a treatment for women who develop mild or moderate depression during the postnatal period. It is now imperative that we investigate whether this professional advice is justified.

The study will contribute towards increasing the evidence base regarding the effectiveness of exercise as a treatment for PND. The intervention may improve the mental health and well-being of women with PND, as well as her child and the family as a whole. Exercise may have additional benefits for women in terms of improving cardiovascular health and reducing weight, which have been identified as particular conerns for new mothers.

Findings of the research will inform future updates of the NICE guidance on antenatal and postnatal mental health, as well as NICE guidance for the management of depression.

### Ethical approval

Favourable ethical opinion for this study was granted by NRES Committee West Midlands-Solihull in August 2009 (09/H1206/94).

## Competing interests

The authors declare they have no competing interests.

## Author contributions

AD wrote the protocol for the study with assistance from KJ, CM, DS, KT, IJ, AR and MM. RB is responsible for coordinating the study and SC is responsible for delivering the intervention. All authors have reviewed and approved the final version of this manuscript.

## Pre-publication history

The pre-publication history for this paper can be accessed here:

http://www.biomedcentral.com/1471-2393/12/45/prepub
